# Optimal calorie restriction threshold: effect of FATmax exercise combined with different proportions of calorie restriction on hypercholesterolemia

**DOI:** 10.3389/fphys.2025.1510949

**Published:** 2025-01-27

**Authors:** Yuetong Wu, Li Zhang, Jing Li, Boyang Xue, Wenyuan Shang, Yingli Lu

**Affiliations:** ^1^ Sports Rehabilitation Research Center, China Institute of Sport Science, Beijing, China; ^2^ College of Education and Sports Science, Yangtze University, Jingzhou, China; ^3^ School of Exercise and Health, Shanghai University of Sport, Shanghai, China

**Keywords:** FATmax exercise, calorie restriction, LDL-C, ApoB, proprotein convertase subtilisin kexin type 9 (PCSK9)

## Abstract

**Background/Objectives:**

To evaluate the impact of maximal fat oxidation intensity exercise combined with calorie restriction intervention on lipid-related parameters in a hypercholesterolemic population, and to determine if an optimal range of calorie restriction exists for effectively enhancing blood lipid profiles.

**Methods:**

A 4-week intervention study combined exercise and calorie restriction for 64 patients aged 18–60 with secondary hypercholesterolemia. Ultimately, 43 participants completed the study. The dietary intervention adhered to the principles of a balanced diet, with meal plans designed to provide three meals per day for the duration of the study. Each subject’s daily calorie intake was set to match their individual resting energy expenditure (REE) plus varying proportions of physical activity (PA) calories. Participants were divided into four groups based on these proportions: REE only, REE + PA33%, REE + PA67%, and REE + PA100%. FATmax exercises were conducted 5 times per week, lasting 1 h each.

**Results:**

1) Compared with baseline, subjects’ body weight, fat mass and body fat rate decreased significantly; fat-free mass also decreased significantly in the REE, REE + PA33%, and REE + PA67% groups. 2) Subjects’ serum TC decreased significantly; serum LDL-C and ApoB decreased significantly in the REE, REE + PA33%, and REE + PA67% groups; there were no significant changes in serum HDL-C and ApoA1. 3) Serum PCSK9 was significantly decreased in the REE and the REE + PA 67% groups; serum LDLR was significantly decreased in all groups of subjects. 4) Between the groups, the rate of change in serum LDL-C was significantly different.

**Conclusion:**

FATmax exercise combined with proper proportions of calorie restriction can significantly decrease serum cholesterol levels and fat mass in hypercholesterolemic patients. Nevertheless, it is misleading to assume that a drastic reduction in calorie intake invariably results in superior outcomes. Optimal cost-effectiveness may be achieved within a calorie restriction range of REE + PA33-67%.

## 1 Introduction

Calorie restriction, involving a 25%–30% reduction in daily calorie intake while ensuring sufficient essential nutrients and avoiding malnutrition, effectively regulates lipids and reduces body weight (Ewa et al., 2022). However, prolonged low-calorie diets or inadequate nutrition may increase skeletal muscle catabolism, impairing muscle function and reducing muscle mass (Kiesswetter, 2017). Recent studies indicate that a moderate 25%–30% calorie reduction can reduce triglyceride and total cholesterol levels while promoting weight loss. Some studies also suggest that more substantial calorie reductions (30%–60%) can significantly lower cholesterol levels and enhance body composition (Napoleão et al., 2021; Mitchell et al., 2016; Martin et al., 2016; Ramos-Campo et al., 2024; Gallardo et al., 2014; Guo et al., 2023).

Exercise is crucial for reducing serum cholesterol and promoting weight management. Maximum fat oxidation intensity exercise is an aerobic exercise with the peak rate of fat oxidation during exercise (JeanFrédéric et al., 2022). Numerous studies have demonstrated that this particular exercise intensity positively influences glucose and lipid metabolism, significantly decreases total serum cholesterol and body fat rate, and is safe and efficient (Dumortier et al., 2003; Heda, 2018; PENG et al., 2022).

Both exercise and diet can reduce blood lipid levels and decrease fat mass. The combination of these interventions is known to yield better results than either alone (Xie et al., 2024). However, research on FATmax exercise with caloric restriction is limited. Previous studies showed that prolonged energy intake reduction may dilute weight loss effect due to endocrine function disorder, energy expenditure reducing, and appetite increase (Ochner et al., 2013). The properties of FATmax exercise, including minimize appetite and highest lipid oxidation rates, can assure its effectiveness on weight loss when combined with adequate protein intake (Brun et al., 2022). Additionally, 4 weeks of FATmax training without calorie restriction is less effective in reducing fat mass compared to 8–10 weeks of training (Chávez-Guevara et al., 2020). In contrast, 16 weeks of FATmax exercise combined with caloric restriction significantly reduced serum cholesterol levels and body weight (Ipavec-Levasseur et al., 2015). Thus, it seems that long time, which is at least 8–10 weeks, is necessary for FATmax exercise to exhibit significant weight loss effect, and even longer time to show blood lipid reduction. Futhermore, the optimal calorie intake for reducing serum cholesterol and body composition remains unclear. So this preliminary investigation intends to provide a more personalized and precise approach for weight loss and lipid reduction, specifically identifying the most effective calorie restriction regimen for reducing serum cholesterol levels and body composition in individuals with secondary hypercholesterolemia,and to provide a reference for optimizing fat loss exercise and diet plans.

## 2 Materials and methods

### 2.1 Study registration and participants

64 subjects with secondary hypercholesterolemia were recruited from Yangtze University (Jingzhou, China). Prior to the intervention, all subjects voluntarily signed the Informed Consent Form and completed the Personal Basic Information Questionnaire, the China Physical Activity Questionnaire (CPAQ), and the Physical Activity Readiness Questionnaire (2014 PAR-Q+).

Inclusion and exclusion criteria for subjects: 1) age 18–60 years; 2) weight fluctuation ≤5 kg in 6 months; 3) secondary hypercholesterolemia, meeting the diagnostic criteria of TC ≥ 5.2 mmol/L or LDL-C ≥3.4 mmol/L as defined in the Chinese Guidelines for Prevention and Control of Dyslipidemia in Adults (2016 Revision); 4) exclusion of those with a history of serious diseases such as severe hypertension, diabetic complications, coronary heart disease of grade 3 or above, etc.; 5) no systematic exercise training or exercise habit in the past 5 years; 6) exclusion of those who are unable to carry out prolonged, low-intensity exercise due to injuries or illnesses.

**FIGURE 1:**
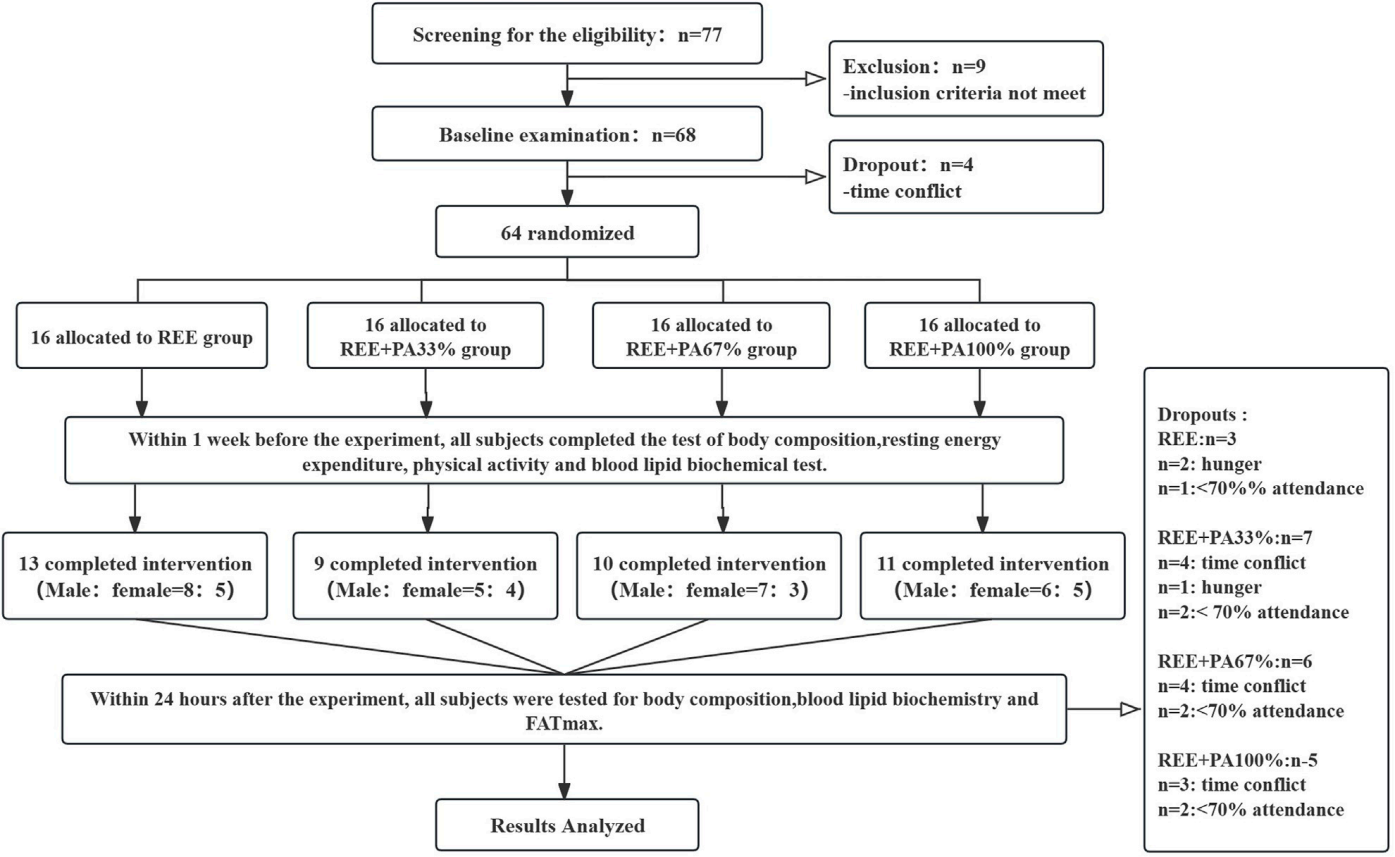
Flow chart of study: Abbreviations: REE, resting energy expenditure; PA, physical activity.

The protocol received approval from the Ethics Committee for Human Exercise Experimentation at the China Institute of Sport Science, with the approval number 2023082401. The experimental procedure is as follows: Figure 1.

### 2.2 Diet and exercise program

#### 2.2.1 Calorie restriction program

Daily dietary calorie intake was calculated as the sum of resting energy expenditure (REE) and physical activity (PA) calories. REE was measured using a gas metabolism analyzer, while PA calories were assessed via accelerometers. Subjects were divided into four groups based on different calorie restriction ratios: 1) REE group (n = 16); 2) REE + PA33% group (n = 16); 3) REE + PA67% group: (n = 16); 4) REE + PA100% group (n = 16). All meals followed a balanced dietary structure in accordance with the Balanced Dietary Guidelines for Chinese Residents (2022). The calorie distribution of the three meals was breakfast: lunch: dinner = 30%: 40%: 30%. The macronutrient energy supply ratio was carbohydrate: protein: fat = 50–60%: 20%–30%: 20%–25%, with other nutrients matched according to the Dietary Reference Intakes for Chinese Residents (DRIs 2013).

Before the experiment, we surveyed subjects’ dietary restrictions and preferences via mobile phone questionnaires to ensure the food matched their tastes. During the experiment, three meals were prepared daily for all participants and meticulously weighed to ensure strict adherence to the protocol. Subjects were required to eat all provided food at each meal and follow a strict three-meal-per-day schedule without additional food. They were encouraged to increase water intake during the intervention period. Most subjects ate on-site, while a few took meals home or to the office. Additionally, we monitored body composition daily by using the InBody 270 to assess the intervention’s effectiveness and prevent any abnormal weight gain due to unauthorized food intake.

#### 2.2.2 Exercise training program

Subjects completed a 4-week exercise intervention program, with each session lasting 1 h and conducted 5 times per week. Given that individuals unaccustomed to regular exercise primarily exhibit adaptive physiological changes characterized by adjustments in target heart rate during the initial training period, all subjects underwent 12 FATmax tests throughout the intervention. The target heart rate corresponding to the measured FATmax was used as the training target heart rate to ensure precise exercise intensity.

The FATmax testing schedule was as follows: before each training session in the first week; before Monday and Thursday sessions in week 2, 3, and 4. A final FATmax test was completed within 1 day post-intervention. Subjects were instructed to maintain continuous exercise for 1 h at the target heart rate, ensuring it remained within ±5 beats of the target. Vigorous physical activity was avoided on weekends.

### 2.3 Measurements

#### 2.3.1 Body composition

Subjects were instructed to fast for a minimum of 8 h and empty bowels. They were also required to wear light clothing and remove metal jewelry for the test. Body composition analyzer (Visbody-D Pro3, China) was used to measure body weight, body fat, fat-free weight, and body fat rate before and after the intervention.

#### 2.3.2 Resting energy expenditure

Preparation conditions for subjects: 1) no vigorous exercise within 24 h before the test, and abstention from beverages containing alcohol, caffeine, or strong tea; 2) maintenance of a normal diet and adequate sleep; 3) fasting for at least 8 h. The testing temperature was maintained at 25°C. Subjects tested quieyly for 15 min to stabilize their heart rate before the test began. Participants wore a gas metabolism analyzer (Metamax 3B, Cortex, Germany) and a Polar heart rate monitor (Polar H7, Finland) while lying supine. During the test, subjects remianed awake, calm,and breath regularly for 20 min. Measurements over 20 min showed the subject’s oxygen uptake remained within a 5% fluctuation for at least five consecutive minutes, indicating stability. The mean value represented the REE. (Sanchez-Delgado et al., 2018).

#### 2.3.3 Physical activity

Accelerometer sensors (ActiGraph wGT3X-BT; ActiGraph LLC, Pensacola, FL) measured Subjects’ daily physical activity levels. Subjects wore the device above the right iliac crest for five consecutive days, excluding sleep and bathing. The average energy expenditure from the three most active days (two weekdays and one weekend day) represented the highest daily physical activity intensity.

#### 2.3.4 Fatmax test

The test was conducted at least 1 h postprandial, using a portable gas metabolism analyzer (Metamax 3B, Cortex, Germany), a Polar heart rate monitor (POLAR H7, Finland), and a treadmill (Mercury 4.0, h/p/cosmos, Germany).

Test procedure: An incrementally loaded treadmill exercise starting at 4 km/h, increasing speed by 1 km/h every 2 min until 6 km/h, then increasing the gradient by 1% every 2 min while monitoring the respiratory quotient.

Criteria for terminating the experiment: The respiratory quotient reached 0.95 (Amaro-Gahete et al., 2019). The fat oxidation rate was calculated as [fat oxidation (g/min) = 1.6946*V0_2_ (L/min)-1.7012*VCO_2_(L/min)]. The highest fat oxidation rate identified as FATmax, with the corresponding heart rate set as the target for exercise.

#### 2.3.5 Blood measurements

Fasting venous blood samples were collected from subjects 48 h before the experiment and 24 h after the fourth week. Serum was extracted for analysis of TC, HDL-C, LDL-C,ApoA1 and ApoB were measured using standardized enzymatic assays on a Chemistry Analyzer (Beckman Coulter AU5800, Brea, CA, USA). Serum levels of PCSK9 and LDLR were quantified by ELISA according to the manufacturer’s instructions (R&D, MULTISKAN MK3, Thermo, United States).

### 2.4 Data analysis

Data were analyzed using SPSS 27.0, with graphs generated in GraphPad Prism 9.0. Data normality was assessed using the Kolmogorov-Smirnov test. Paired t-tests were used for within-group comparisons, while one-way ANOVA or the Kruskal–Wallis test was applied for between-group differences based on data distribution. To control the error rate at 5%, Bonferroni correction (for homogeneous variances) or Tamhane’s T2 test (for heterogeneous variances) was used. Statistical significance was set at P < 0.05, with highly significant differences indicated by P < 0.01.

## 3 Results

### 3.1 Changes in body composition

**FIGURE 2:**
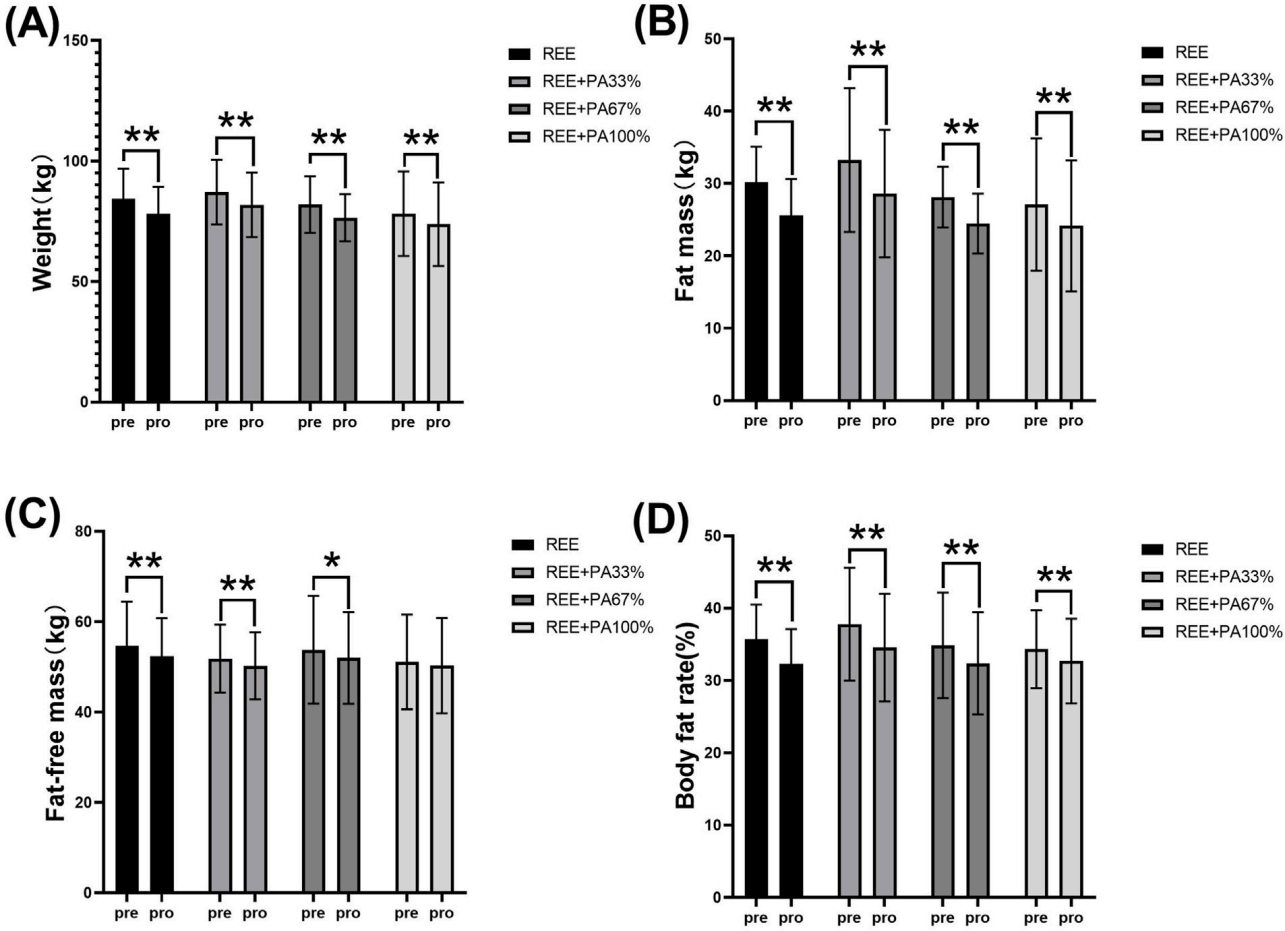
Changes in body weight **(A)**, fat mass **(B)**, fat-free mass **(C)**, and body fat rate **(D)** pre and post the intervention * represents p < 0.05; ** represents p < 0.01.

Table 1 and Figure 2 illustrate the extent of changes in body composition relative to baseline. Compared with the baseline, the body weight of the subjects decreased significantly after 4 weeks. Except for the REE + PA100% group, the fat-free mass of the other groups decreased significantly. The fat mass of four groups decreased significantly. The body fat rate of the four groups also decreased significantly.

**TABLE 1:** Changes in body composition, lipids, PCSK9 and LDLR levels in subjects within each group before and after the intervention.

Variable	Group	N	Pre-test	Post-test	Improvement (pre-test to post-test)		
			M ± SD	M ± SD	Mean differences	*p*	d
Weight (kg)	REE	13	84.42 ± 12.36	78.08 ± 11.18	6.34	0.001^**^	2.70
	REE + PA33%	9	87.14 ± 13.4	81.8 ± 13.38	5.34	0.001^**^	2.26
	REE + PA67%	10	81.93 ± 11.75	76.48 ± 9.82	5.45	0.001^**^	2.22
	REE + PA100%	11	78.2 ± 17.5	73.81 ± 17.27	4.39	0.001^**^	3.29
Fat-free mass (kg)	REE	13	54.62 ± 9.8	52.37 ± 8.43	2.25	0.002^**^	1.08
	REE + PA33%	9	51.82 ± 7.55	50.24 ± 7.41	1.58	0.003^**^	1.40
	REE + PA67%	10	53.79 ± 11.92	52.0 ± 10.14	1.79	0.034	0.79
	REE + PA100%	11	51.1 ± 10.46	50.3 ± 10.51	0.80	0.053	0.66
Fat mass (kg)	REE	13	30.15 ± 4.96	25.58 ± 5.04	4.57	0.001^**^	2.29
	REE + PA33%	9	33.24 ± 9.94	28.62 ± 8.83	4.62	0.002^**^	1.56
	REE + PA67%	10	28.12 ± 4.19	24.48 ± 4.13	3.64	0.001^**^	4.57
	REE + PA100%	11	27.1 ± 9.14	24.15 ± 9.06	2.95	0.001^**^	1.80
Body fat rate (kg)	REE	13	35.7 ± 4.8	32.28 ± 4.84	3.42	0.001^**^	2.85
	REE + PA33%	9	37.79 ± 7.81	34.56 ± 7.43	3.23	0.003^**^	1.38
	REE + PA67%	10	34.88 ± 7.29	32.39 ± 7.06	2.49	0.001^**^	2.09
	REE + PA100%	11	34.34 ± 5.38	32.69 ± 5.86	1.65	0.003^**^	1.17
TC (mmol/L)	REE	13	5.71 ± 0.56	4.66 ± 0.63	1.05	0.001^**^	1.46
	REE + PA33%	9	5.79 ± 0.64	4.92 ± 0.76	0.88	0.001^**^	2.87
	REE + PA67%	10	6.03 ± 0.88	5.07 ± 0.76	0.96	0.004^**^	1.23
	REE + PA100%	11	5.87 ± 0.65	5.37 ± 0.77	0.50	0.043^*^	0.70
HDL-C (mmol/L)	REE	13	1.29 ± 0.12	1.22 ± 0.15	0.07	0.143	0.44
	REE + PA33%	9	1.32 ± 0.19	1.28 ± 0.2	0.05	0.317	0.36
	REE + PA67%	10	1.36 ± 0.23	1.3 ± 0.24	0.05	0.385	0.29
	REE + PA100%	11	1.27 ± 0.21	1.29 ± 0.27	−0.02	0.758	−0.10
LDL-C (mmol/L)	REE	13	3.68 ± 0.35	2.86 ± 0.46	0.81	0.001^**^	1.81
	REE + PA33%	9	3.59 ± 0.64	3.04 ± 0.54	0.55	0.001^**^	1.87
	REE + PA67%	10	3.72 ± 0.65	3.02 ± 0.52	0.71	0.001^**^	1.70
	REE + PA100%	11	3.53 ± 0.47	3.29 ± 0.52	0.24	0.146	0.48
ApoA1 (g/L)	REE	13	1.29 ± 0.14	1.22 ± 0.11	0.10	0.052	0.60
	REE + PA33%	9	1.45 ± 0.25	1.36 ± 0.2	0.10	0.126	0.57
	REE + PA67%	10	1.34 ± 0.21	1.31 ± 0.24	0.03	0.367	0.30
	REE + PA100%	11	1.35 ± 0.23	1.35 ± 0.27	−0.00	0.985	−0.01
ApoB (g/L)	REE	13	1.25 ± 0.14	1.01 ± 0.16	0.24	0.001^**^	1.54
	REE + PA33%	9	1.29 ± 0.25	1.13 ± 0.22	0.17	0.001^**^	1.63
	REE + PA67%	10	1.26 ± 0.22	1.06 ± 0.23	0.21	0.003^**^	1.26
	REE + PA100%	11	1.24 ± 0.27	1.12 ± 0.19	0.12	0.075	−0.60
PCSK9(ng/mL)	REE	13	189.5 ± 45.62	141.3 ± 44.22	48.20	0.002^**^	1.37
	REE + PA33%	9	211.85 ± 59.54	163.49 ± 66.46	48.36	0.072	0.82
	REE + PA67%	10	201.14 ± 48.45	163.74 ± 47.78	37.40	0.029^*^	0.89
	REE + PA100%	11	167.35 ± 48.99	160.72 ± 50.56	6.63	0.716	0.11
LDLR (ng/mL)	REE	13	70.62 ± 33.73	38.9 ± 10.26	31.72	0.018^*^	0.91
	REE + PA33%	9	61.82 ± 21.35	42.93 ± 17.45	18.89	0.004^**^	1.70
	REE + PA67%	10	68.45 ± 22.2	38.76 ± 12.71	29.69	0.001^**^	1.60
	REE + PA100%	11	88.83 ± 63.26	57.6 ± 48.47	31.22	0.001^**^	1.80

Note: Values are presented as Mean ± SD.

Abbreviations: TC, total cholesterol; HDL-C, high density lipoprotein cholesterol; LDL-C, low density lipoprotein cholesterol; ApoA1, apolipoprotein A1; ApoB, apolipoprotein B; PCSK9, proprotein convertase subtilisin/kexin type 9; LDLR, low density lipoprotein receptor.

p < 0.05 indicated that there was significant difference before and after the experiment.

**FIGURE 3:**
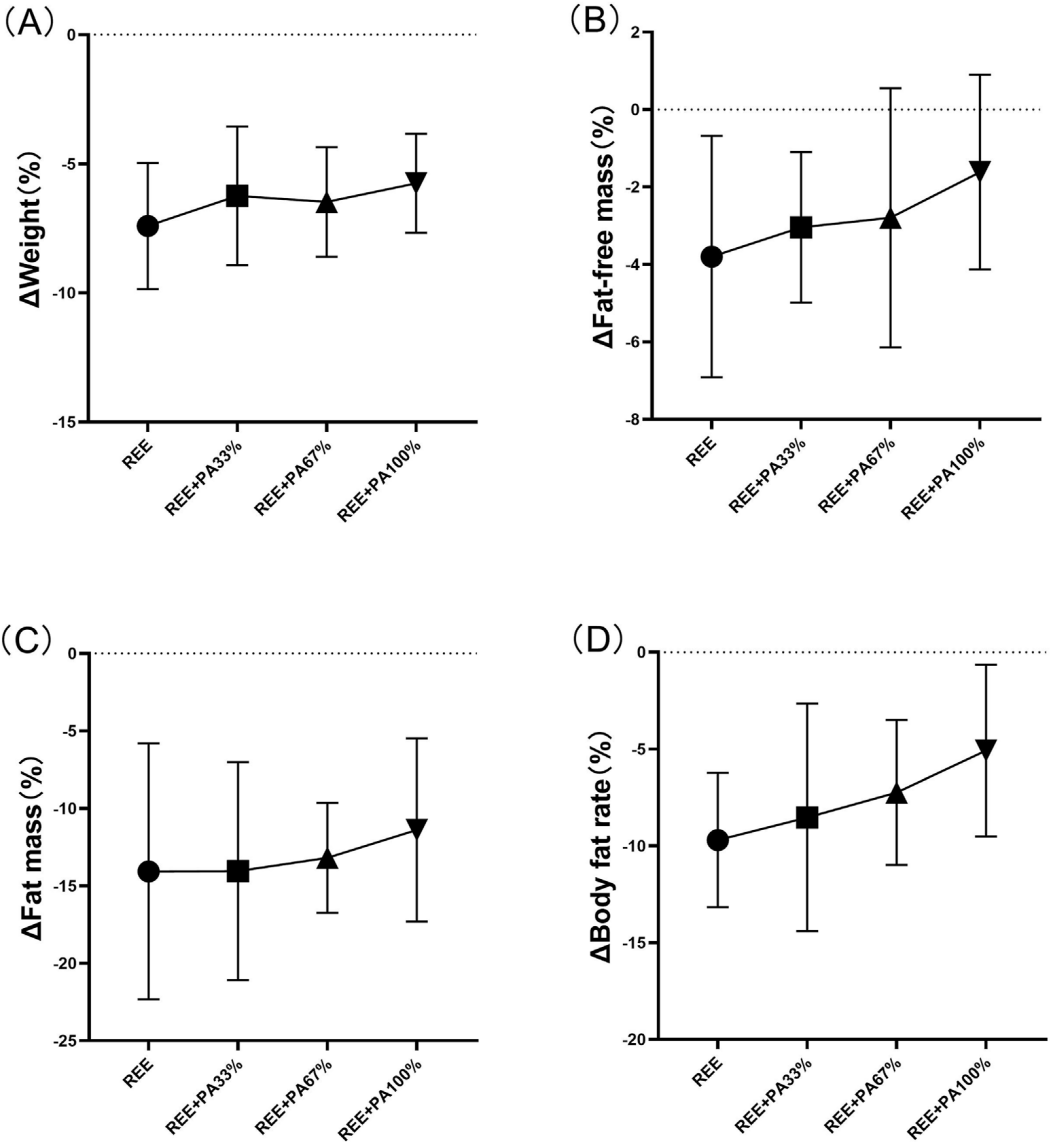
Rates of change between groups in body weight **(A)**, fat mass **(B)**, fat-free mass **(C)**, and body fat rate **(D)**: Circles represent the rate of change in the REE group; squares represent the rate of change in the REE + PA33% group; square triangles represent the rate of change in the REE + PA67% group; and inverted triangles represent the rate of change in the REE + PA100% group. Δ (%) = (after experiment-before experiment)/before experiment × 100%.

The body composition measures for all groups are summarized in Table 2 and Figure 3, which highlight the statistical differences among groups. No significant differences were observed in the rate of change of body weight, fat-free mass, fat massand body fat rate among the four groups.

**TABLE 2:** Rates of change in body composition, lipids, PCSK9 and LDLR levels between groups**.**

	Groups								
	REE (n = 13)	REE + PA33% (n = 9)	REE + PA67% (n = 10)	REE + PA100% (n = 11)	F	p			η2	
ΔWeight (%)	−7.41 ± 2.45	−6.24 ± 2.68	−6.48 ± 2.12	−5.76 ± 1.92	1.095	0.363			0.078	
ΔFat-free mass (%)	−3.8 ± 3.12	−3.04 ± 1.94	−2.79 ± 3.35	−1.61 ± 2.51	1.205	0.321			0.085	
ΔFat mass (%)	−14.06 ± 8.26	−14.04 ± 7.04	−13.19 ± 3.56	−11.39 ± 5.91	0.405	0.750			0.030	
ΔBody fat rate (%)	−9.7 ± 3.47	−8.53 ± 5.88	−7.24 ± 3.74	−5.08 ± 4.44	2.378	0.085			0.155	
ΔTC (%)	−17.93 ± 10.99	−15.39 ± 5.75	−15.22 ± 11.88	−8.24 ± 12.06	1.742	0.174			0.118	
ΔHDL-C (%)	−5.21 ± 12.62	−3.93 ± 11.93	−2.86 ± 10.87	1.33 ± 13.24	0.608	0.614			0.045	
ΔLDL-C (%)	−21.91 ± 11.27	−15.05 ± 7.57	−18.47 ± 10.57	−6.29 ± 14.12^##^	4.060	0.013^&^			0.238	
ΔApoA1 (%)	−6.65 ± 11.81	−6.53 ± 12.26	−2.4 ± 7.16	0.22 ± 11.25	1.045	0.383			0.074	
ΔApoB (%)	−18.69 ± 11.1	−12.72 ± 8.22	−16.35 ± 13.62	−7.93 ± 15.77	1.600	0.205			0.110	
ΔPCSK9(%)	−25.15 ± 17.77	−20.37 ± 26.05	−17.39 ± 20.26	0.36 ± 35.28	1.883	0.152			0.146	
ΔLDLR (%)	−36.46 ± 25.31	−30.02 ± 14.37	−41.34 ± 15.73	−37.48 ± 11.19	0.552	0.650			0.048	

Note: Values are expressed as Mean ± SD of rate of change.

Δ (%) = (after experiment-before experiment)/before experiment × 100%.

^&^represents p < 0.05 indicated that there was significant difference between groups; ^##^represents p < 0.01 indicated that there was significant difference between REE and REE + PA100% groups.

### 3.2 Changes in lipid

**FIGURE 4:**
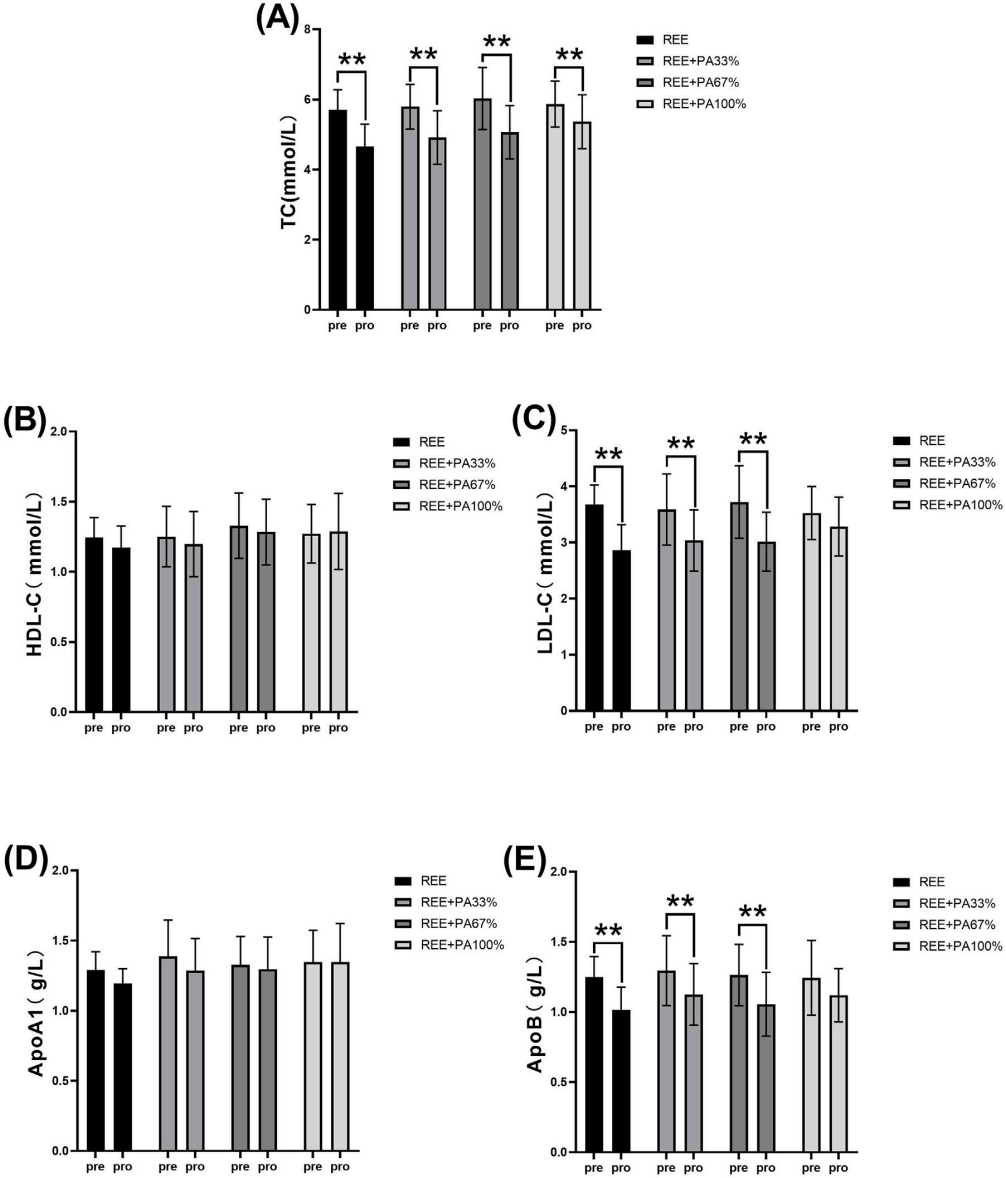
Changes in serum TC **(A)**, HDL-C **(B)**, LDL-C **(C)**, ApoA1 **(D)**, and ApoB **(E)** pre and post the intervention. * represents p < 0.05; ** represents p < 0.01.

Table 1 and Figure 4 illustrates the magnitude of changes in blood lipid relative to baseline. Compared with the baseline, the serum TC of the subjects significantly decreased after 4 weeks. There was no significant change in serum HDL-C in all four groups; there was also no significant change in serum ApoA1. Except for the REE + PA100% group, the serum LDL-C in the REE, REE + PA33% and REE + PA67% groups all decreased significantly. Except for the REE + PA100% group, the serum ApoB in the REE, REE + PA33% and REE + PA67% groups all decreased significantly.

**FIGURE 5:**
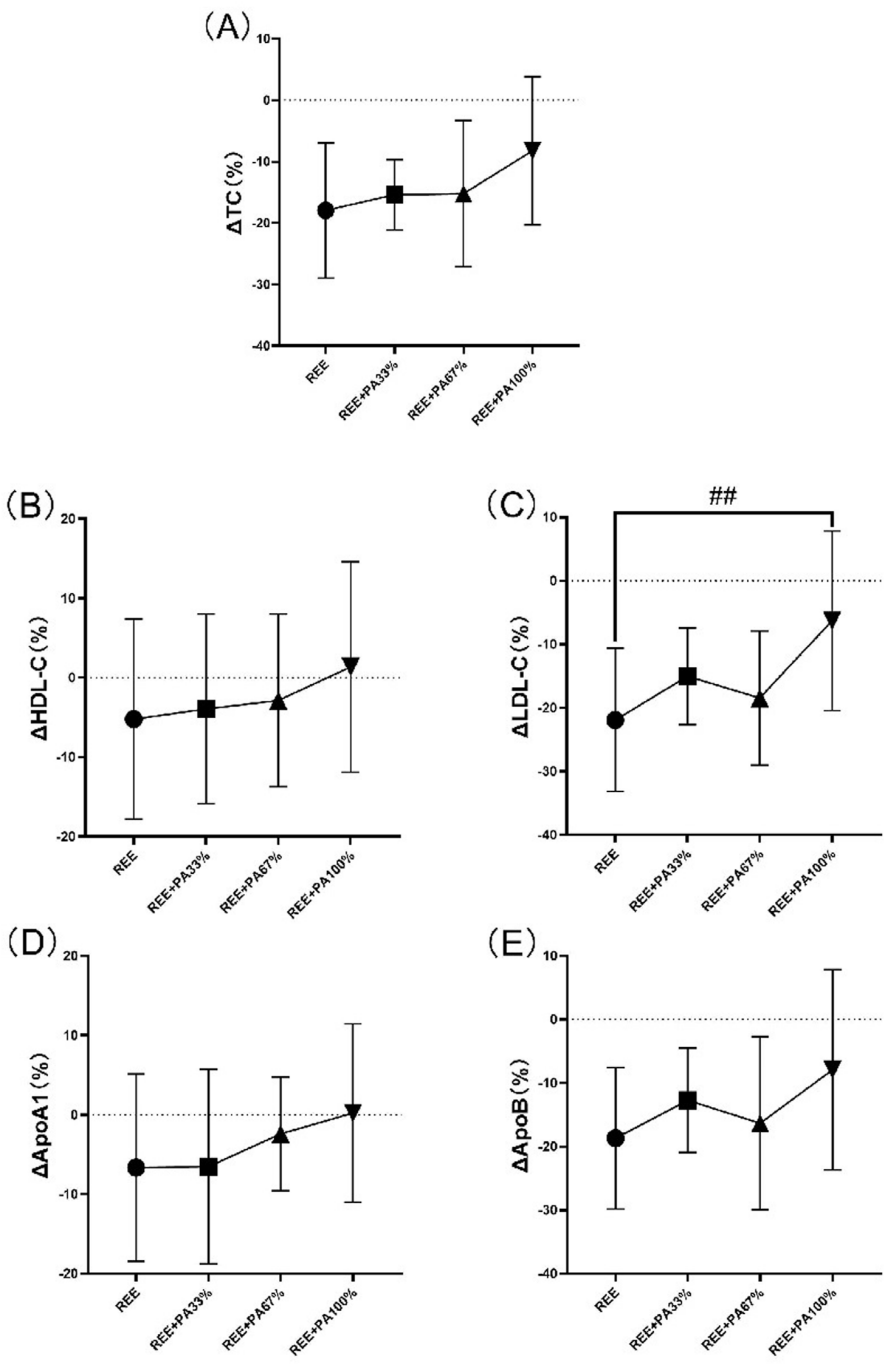
Rates of change between groups in serum TC **(A)**, HDL-C **(B)**, LDL-C **(C)**, ApoA1 **(D)**, and ApoB **(E)**: Circles represent the rate of change in the REE group; squares represent the rate of change in the REE + PA33% group; square triangles represent the rate of change in the REE + PA67% group; and inverted triangles represent the rate of change in the REE + PA100% group. Δ(%) = (after experiment-before experiment)/before experiment × 100%. ## represents p < 0.01 indicated that there was significant difference between REE and REE + PA100% groups.

The blood lipid measurements for all groups are presented in Table 2 and Figure 5, illustrating the statistical differences among groups. No significant differences were observed in the rates of change for serum TC, HDL-C, ApoA1, and ApoB across the groups. However, the rate of change for serum LDL-C was statistically significant among groups, particularly in the REE group and the REE + PA100% group (p = 0.01). Specifically, 77% of participants in the REE group, 56% in the REE + PA33% group, 60% in the REE + PA67% group, and 27% in the REE + PA100% group exhibited a reduction in serum TC levels below the inclusion criteria. Furthermore, 85% of participants in the REE group, 67% in the REE + PA33% group, 70% in the REE + PA67% group, and 45% in the REE + PA100% group demonstrated a decrease in serum LDL-C levels below the inclusion criteria.

### 3.3 Changes in serum PCSK9 and LDLR

**FIGURE 6:**
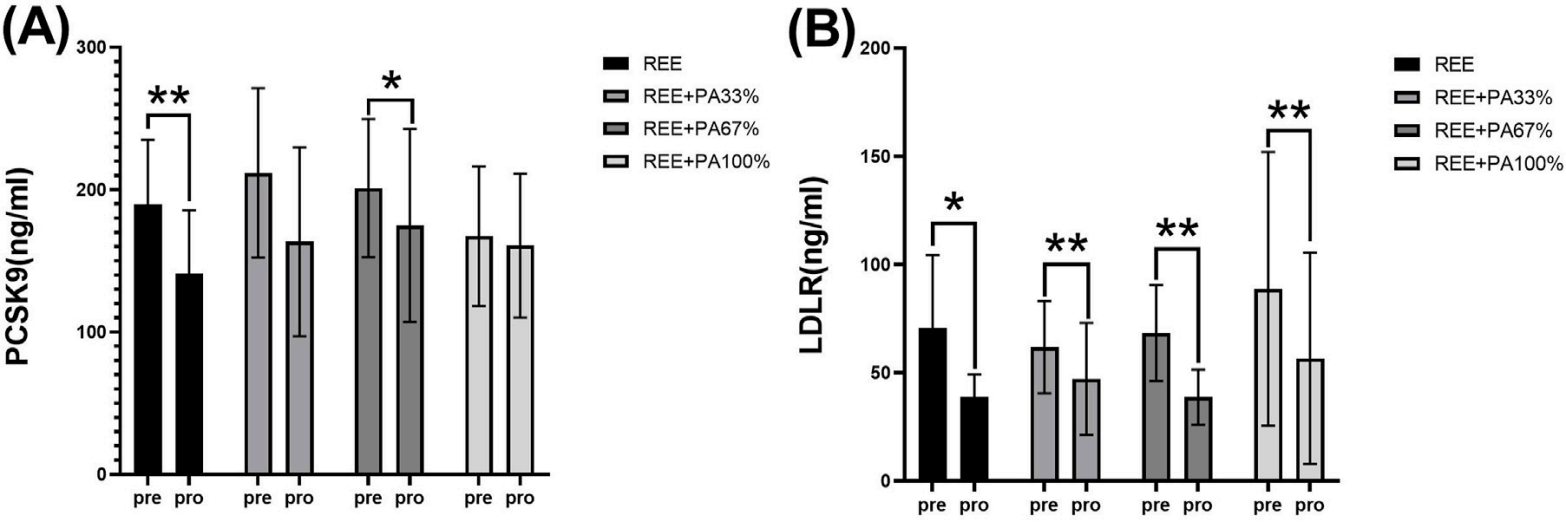
Changes in serum PCSK9 **(A)** and LDLR **(B)** pre and post the intervention * represents p < 0.05; ** represents p < 0.01.

As illustrated in Table 1 and Figure 6, serum PCSK9 levels were significantly reduced in the REE group and the REE + PA67% group compared to the baseline. In contrast, no significant changes were observed in the remaining two groups. Serum LDLR levels decreased significantly across all four groups.

**FIGURE 7:**
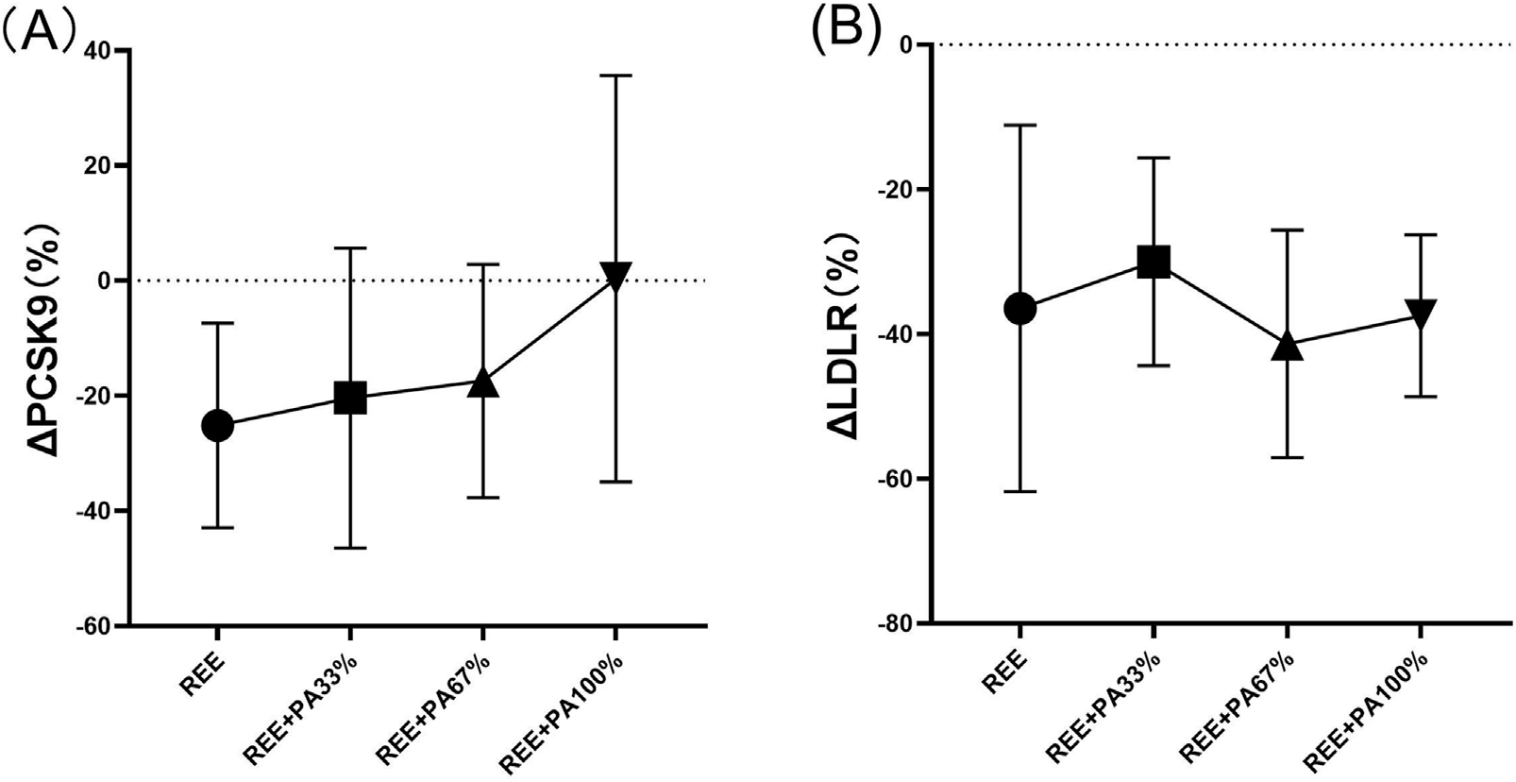
Rates of change between groups in serum PCSK9 **(A)** and LDLR **(B)**: Circles represent the rate of change in the REE group; squares represent the rate of change in the REE + PA33% group; square triangles represent the rate of change in the REE + PA67% group; and inverted triangles represent the rate of change in the REE + PA100% group. Δ (%) = (after experiment-before experiment)/before experiment × 100%.

The differences in PCSK9 and LDLR levels among the groups are summarized in Table 2 and Figure 7. However, the rate of change in serum PCSK9 and LDLR levels did not reach statistical significance.

## 4 Discussion

### 4.1 Effects of FATmax exercise combined with calorie restriction on body composition

The exercise intensity at which fat oxidation peaks during exercise is called FATmax (Purdom et al., 2018). FATmax exercise improve lipid metabolism, promote fatty acid transport and oxidation, improve body composition, and increase cardiorespiratory endurance in overweight and obese individuals (Cao et al., 2019; Dizhi et al., 2023; Jiang et al., 2020). Calorie restriction also helps lower blood lipid levels, reduce body weight and decrease cardiovascular risk (Luna-Castillo et al., 2022; Wang et al., 2024; Smith et al., 2024; Son et al., 2021; Senesi et al., 2021). In an animal study, a 45% calorie restriction led to significantly greater reductions in body weight and visceral fat compared to a 25% restriction (Yu et al., 2018). Calorie restriction lowers body weight by decreasing daily intake; combining it with exercise further enhance this effect (Xie et al., 2024). Theoretically, FATmax indicates the exercise intensity for optimal fat loss. However, in practice, individual differences in glycolipid metabolism and nutritional status can affect FATmax measurement results.

This study found that 4 weeks of FATmax exercise with varying degrees of calorie restriction can significantly reduce body weight, fat mass, and body fat rate in individuals with hypercholesterolemia (Figure 2). Previous studies on FATmax training without dietary intervention typically lasted 2–4 months. This study, however, applied a 4-week FATmax training combined with caloric restriction, leading to significant reductions in weight and fat mass. This highlights the effectiveness of combining precise FATmax exercise with proper diet control for fat loss and blood lipids reduction (Chávez-Guevara et al., 2020; Donglei et al., 2025; Tan et al., 2016). This study found no statistically significant differences in body composition change among the four groups, likely due to the limited sample size and short intervention duration (Figure 3). However,The trend in body composition changes across the four groups shows that the rate of fat mass reduction follows a curvilinear downward pattern as calorie intake decreases, with the inflection point occurring between REE + PA33% and 67% (Figure 3C). Similarly, the inflection points for body weight and fat-free mass changes also fall within this range (Figures 3A, B). The study reveals significant changes in fat-free mass in three groups, indicating that lower calorie intake does not always lead to better health. Previous research has also observed lean body mass loss during fat loss (Claire et al., 2020). Therefore, it is advisable to pursue fat loss through short-term rather than long-term calorie restriction in order to minimize excessive lean body mass loss.

### 4.2 Effects of FATmax exercise combined with calorie restriction on blood lipids

Regarding hypercholesterolemia, particular attention should be given to alterations in LDL-C levels, as elevated LDL-C is a primary contributor to an increased risk of cardiovascular disease. (JianJun et al., 2023). Previous research shows that exercise and calorie restriction significantly affect lipid profiles, while cholesterol levels are mainly influenced by diet (Son et al., 2021; JianJun et al., 2023; Tianxiao et al., 2022; Napoleão et al., 2021). FATmax exercise also significantly improves metabolic syndromes, including dyslipidemia (Sijie et al., 2018). Exercise can enhance the uptake and utilization of free fatty acids from the blood by active skeletal muscles, consequently reducing LDL-C and cholesterol levels (Fanming et al., 2022). Yingying Li et al. showed that FATmax exercise significantly reduces LDL-C levels, potentially lowering the risk of coronary heart disease (Yingying et al., 2018). Consequently, a scientific exercise regimen and balanced diet can help patients manage their lipid profiles effectively. Combining FATmax exercise with calorie restriction may be more effective in reducing cholesterol levels.

This study found a significant reduction in serum TC across all groups. Except for the REE + PA100% group, the other three groups showed significant decreases in serum LDL-C and ApoB. No significant changes were observed in serum HDL-C and ApoA1 (Figure 4). Previous studies showed that FATmax exercise significantly decreased LDL-C but not TC when used alone. In contrast, this study found significant decreases in both LDL-C and TC (Donglei et al., 2025; Chávez-Guevara et al., 2020). This highlights the importance of accurate FATmax intensity settings and proper calorie restriction. Most prior studies measured FATmax only once before the experiment, leading to inaccurate intensity levels over time. In this study, FATmax was repeatedly measured during the experimental period to ensure training accuracy, better reflecting its true effect on fat loss and lipid reduction. Additionally, this study is the first to observe the combined effects of different levels of dietary calorie restriction and FATmax.

The between-groups analysis showed a significant difference in LDL-C change rate between the REE and REE + PA100% groups (Figure 5C), while no significant different was observed between REE + PA33%-67% groups. This indicates that LDL-C changes are influenced by caloric intake, while more pronounced caloric restriction was required for statistically significant reductions within short-term. The rates of change of serum TC, HDL-C, and ApoA1 decreased curvilinearly with lower calorie intake but showed a plateau trend in the range of REE + PA33%-67% (Figures 5A, B, D). Recent studies have shown that ApoA1 and ApoB are more accurate predictors of cardiovascular disease risk (Pikkemaat et al., 2024). Inflection points in serum LDL-C and ApoB change curves were observed within the range of REE + PA33%-67%, indicating synchronized trends (Figures 5C, E). It suggests that calorie restriction may affect ApoB levels. Additionally, calorie restriction may also alter HDL-C and LDL-C levels by enhancing ApoA1 function or expression, decreasing ApoB synthesis, or increasiing its degradation.

### 4.3 Effect of FATmax exercise combined with calorie restriction on serum PCSK9, LDLR

To further investigate the potential mechanisms regulating ApoB and other lipid metabolism pathways, this study examined serum PCSK9 and LDLR levels. PCSK9 and LDLR are key components in lipid metabolism, and their interaction can increase LDL-C levels, raising cardiovascular risk. This mechanism is a significant factor in the pathogenesis and progression of hypercholesterolemia (Vincenzo et al., 2023). Additionally, PCSK9 affects other lipid pathways, such as correlating positively with LDL-C and TG levels in fasting plasma (Cariou et al., 2011; Araki et al., 2014; Cui et al., 2010).

The three genes, PCSK9, LDLR, and ApoB, are intricately involved in LDL metabolism. ApoB serves as the primary protein component of LDL particles. The interaction between PCSK9 and ApoB can inhibit the degradation of ApoB via the autophagosome-lysosome pathway, leading to increased hepatic synthesis of ApoB. Consequently, this results in elevated levels of LDL, TC, and TG (Sun et al., 2012; Soutar, 2011; Rämö et al., 2024). However, calorie restriction effectively reduces PCSK9 levels and inhibits the synthesis of ApoB, LDL-C, and TC, reducing their concentrations. This finding is consistent with the results observed in both the REE group and the REE + PA67% group in this study (Figure 6A) (HUSAM et al., 2018). Furthermore, this study observed that serum PCSK9 levels decreased with reduced calorie intake, similar to changes in TC and LDL-C (Figure 7A). It is hypothesized that at the FATmax exercise intensity, alterations in PCSK9, blood lipids, and fat mass may be closely associated with dietary calorie intake. Although no significant differences were noted in the change rate of LDLR between the four groups, an inflection point was observed in REE + PA 33%–67% group (Figure 7B). Previous research has shown that inhibiting PCSK9 activity can elevate LDLR levels, which contrasts with the reduction observed in our study (Figure 6B). A explanation for this discrepancy may be that the combination of calorie restriction and FATmax exercise reduces PCSK9 levels, thereby decreasing its binding to LDLR. This increases available LDLRs for LDL-C clearance. Consequently, the serum LDLR levels are lower, while LDL-C levels are significantly reduced (Tanja et al., 2013).

Currently, research on the effects of calorie restriction and FATmax exercise on PCSK9 and LDLR is limited. Long-term intervention combining calorie restriction with FATmax exercise can regulate PCSK9 and LDLR through multiple pathways. FATmax exercise promotes adipose tissue catabolism, leading to a significant release of fatty acids into the bloodstream for oxidative metabolism in tissues such as the liver. Concurrently, signals from fatty acid oxidation influence the activity of the liver X receptor (LXR). LXR forms a heterodimer with retinoid X receptor and binds to the LXR response element in the PCSK9 gene promoter. As cholesterol levels rise, PCSK9 expression increases (Wang and Tontonoz, 2018). While calorie restriction combined with FATmax exercise can reduce cholesterol levels, it thereby alters this regulatory balance and inhibits PCSK9 expression. Furthermore, the energy deficit induced by calorie restriction triggers a cascade of metabolic regulatory mechanisms (Lagace, 2014). For instance, the activation of the AMPK signaling pathway decreases mTORC1 activity, consequently suppressing PCSK9 and LDLR-associated gene expression (Author Anonymous, 2024). Furthermore, calorie restriction also decreases the synthesis of PCSK9 by inhibiting sterol regulatory element-binding protein-2(SREBP-2) activity (Horton et al., 2003). Therefore, alterations in calorie restriction proportions may influence PCSK9 and LDLR levels via the SREBP pathway (Ma et al., 2023). Moreover, long-term calorie restriction combined with FATmax exercise may can consistently reduce the blood lipids, reducing the risk of hyperlipidemia.

### 4.4 Optimal calorie restriction range for reducing cholesterol levels

Previous studies show limited effects of dietary control alone on reducing blood lipids (Yong and Zhijun, 2013). In this study, we employed a FATmax exercise regimen combined with varying degrees of calorie restriction. Results indicated that serum TC, LDL-C, and ApoB levels decreased as calorie intake reduced. However, this reduction followed a non-linear trend with inflection points occurring within the range of REE + PA33%-67% group (Figure 5). From the perspective of changes in primary outcome indicators, a greater proportion of subjects in the REE + PA67% group achieved normalization of their TC and LDL-C levels post-intervention compared to those in the REE + PA33% group. This study also examined changes in weight, fat mass, and fat-free mass among the subjects. The findings indicated that when calorie intake was lower than the REE + PA 33% threshold, there was a reduction in weight, but the rate of fat-free mass loss accelerated while fat mass reduction decelerated (Figures 3A–C). In conjunction with the subjects’ acceptance of the experimental protocol during actual intervention, it was observed that the withdrew proportion of participants in each group increased as calorie intake decreased. This trend may be due to the increased sensation of hunger caused by excessive caloric restriction (Table 3). These findings further suggest that very low calorie intake may not be beneficial in practice. For participants, a calorie intake higher than REE is necessary for the sustainability of fat loss program. Furthermore, we observed that serum PCSK9 exhibited a comparable inflection point trend to serum TC (Figure 7A). This suggests that both PCSK9 levels and changes in cholesterol are influenced by calorie intake. Thus, alterations in PCSK9 may represent one of the mechanisms through which exercise with calorie restriction modulates lipid profiles. PCSK9 exhibits stable changes as an upstream regulator. LDLR, acting as an intermediate component, may be influenced by other pathways. Thus, an optimal threshold for calorie restriction may exist to elicit gene expression changes. In summary, calorie restriction within the range of REE + PA33%-67% may exist the most cost-effectiveness health outcomes.

**TABLE 3:** The above figure shows the percentage of people who quit because of hunger and insufficient participation in the total number of people in each group**.**

	REE	REE + PA33%	REE + PA67%	REE + PA100%
Dropout rate	18.75%	18.75%	12.5%	12.5%

Theoretically, short-term interventions combining calorie restriction with FATmax exercise have shown benefits for blood lipids and fat mass. Long-term integration of these approaches will further influence physiological regulation mechanism related to lipid metabolism. Concurrently, exercise may stimulate adipose tissue to secrete beneficial adipokines such as adiponectin, enhancing fatty acid oxidation and insulin sensitivity, and improving lipid metabolism (Zhu et al., 2020). In the long run, these changes will positively impact cardiovascular risk by stabilizing vascular endothelial function and reducing the risk of cardiovascular diseases (Thonusin et al., 2022; García-Prieto and Fernández-Alfonso, 2016).

The FATmax test, used to assess maximum fat oxidation, has been validated for reliability and accuracy in recent studies. Research shows a low coefficient of variation with incremental testing, indicating high test-retest reliability (Maunder et al., 2018). The test also demonstrates consistency across individuals with different training statuses, supporting its clinical utility (Chrzanowski-Smith et al., 2020). Recent studies show that fat oxidation rates may be higher at night than in the morning (Rubio-Valles et al., 2024). However, other research indicates that morning exercise significantly boosts fat oxidation, especially in women (Iwayama et al., 2017). Therefore, there is ongoing debate about how exercise timing affects fat oxidation. Due to equipment limitations, participants were not tested at the same time. While precise protocols minimized FATmax value deviations, future research should explore time-of-day effects on fat oxidation mechanisms and validate results with larger sample sizes. Additionally, this study observed a trend in FATmax heart rate changes while monitoring fat oxidation. This suggests that FATmax heart rate variations may be closely associated with fat oxidation and other training response. Therefore, future research should explore the relationship between heart rate and fat oxidation to optimize exercise prescription design.

This study demonstrates that precise exercise and dietary regimens can significantly enhance intervention efficacy while minimizing individual variability. However, factors such as age and gender may still influence the research results. Future studies should further investigate the effects of these variables on the combination of caloric restriction and FATmax exercise to ensure the robustness and accuracy of the findings. In future clinical practice, it is recommended to conduct thorough personalized assessments of each subject and develop precise dietary plans to ensure adequate calorie intake and balanced nutrition. This approach will prevent metabolic disorders caused by insufficient calorie intake, thereby enhancing the feasibility and effectiveness of the intervention plans. For FATmax exercise, it is advisable to use wearable devices to monitor heart rate, ensuring the precision of exercise intensity. Despite the challenges in providing precise measurements of exercise and resting metabolic rates for a large population, this study suggests adopting a “low-intensity aerobic exercise combined with mild to moderate calorie restriction” program. This approach can effectively reduce fat while preserving fat-free mass to the greatest extent possible. For individuals with obesity and hyperlipidemia who have experienced prolonged difficulties in fat loss, their unique physical fitness and metabolic profiles may require personalized interventions. Through targeted testing, we can design precisely tailored programs to assist them in achieving effective fat loss and lipid reduction.

## 5 Conclusion

FATmax exercise combined with proper proportions of calorie restriction can significantly decrease serum cholesterol levels and fat mass in hypercholesterolemic patients. Nevertheless, it is misleading to assume that a drastic reduction in calorie intake invariably results in superior outcomes. Optimal cost-effectiveness may be achieved within a calorie restriction range of REE + PA33-67%.

## Data Availability

The raw data supporting the conclusions of this article will be made available by the authors, without undue reservation.

## References

[B1] Amaro-Gahete F. J. Sanchez-Delgado G. Helge J. W. Ruiz J. R. (2019). Optimizing maximal fat oxidation assessment by a treadmill-based graded exercise protocol: when should the test end? Front. Physiol. 10, 909. 10.3389/fphys.2019.00909 31396095 PMC6664289

[B2] Araki S. Suga S. Miyake F. Ichikawa S. Kinjo T. Yamamoto Y. (2014). Circulating PCSK9 levels correlate with the serum LDL cholesterol level in newborn infants. Early Hum. Dev. 90, 607–611. 10.1016/j.earlhumdev.2014.07.013 25134067

[B3] Author Anonymous (2024). Department Of Physiology, Jinan University. Sheng li xue bao Acta Physiol. Sin. 72, 371–381.

[B4] Brun J. F. Myzia J. Varlet-Marie E. Raynaud DE Mauverger E. Mercier J. (2022). Beyond the calorie paradigm: taking into account in practice the balance of fat and carbohydrate oxidation during exercise? Nutrients 14, 1605. 10.3390/nu14081605 35458167 PMC9027421

[B5] Cao L. Jiang Y. Li Q. Wang J. Tan S. (2019). Exercise training at maximal fat oxidation intensity for overweight or obese older women: a randomized study. J. Sports Sci. Med. 18, 413–418. 31427862 PMC6683615

[B6] Cariou B. May C. L. Costet P. (2011). Clinical aspects of PCSK9. Atherosclerosis 216, 258–265. 10.1016/j.atherosclerosis.2011.04.018 21596380

[B7] CháVEZ-Guevara I. A. Urquidez-Romero R. PéREZ-LeóN J. A. GonzáLEZ-RodríGUEZ E. Moreno-Brito V. Ramos-JiméNEZ A. (2020). Chronic effect of fatmax training on body weight, fat mass, and cardiorespiratory fitness in obese subjects: a meta-analysis of randomized clinical trials. Int. J. Environ. Res. Public Health 17, 7888. 10.3390/ijerph17217888 33126461 PMC7663534

[B8] Chrzanowski-Smith O. J. Edinburgh R. M. Thomas M. P. Haralabidis N. Williams S. Betts J. A. (2020). The day-to-day reliability of peak fat oxidation and FAT(MAX). Eur. J. Appl. Physiol. 120, 1745–1759. 10.1007/s00421-020-04397-3 32488584 PMC7340634

[B9] Claire L. Isabelle D. G. Dominique L. Isabelle H. Cedric M. (2020). Influence of acute and chronic exercise on abdominal fat lipolysis: an update. Front. Physiology 11, 575363. 10.3389/fphys.2020.575363 PMC7750473 33364972

[B10] Cui Q. Ju X. Yang T. Zhang M. Tang W. Chen Q. (2010). Serum PCSK9 is associated with multiple metabolic factors in a large Han Chinese population. Atherosclerosis 213, 632–636. 10.1016/j.atherosclerosis.2010.09.027 21040917

[B11] Dizhi W. Peizhen Z. Jin L. (2023). Crossover point and maximal fat oxidation training effects on blood lipid metabolism in young overweight women: a pilot study. Front. physiology 14, 1190109. 10.3389/fphys.2023.1190109 PMC10311904 37398909

[B12] Donglei L. Sijie T. Fengying Y. (2025). The improvement of maximal fat oxidation intensity on body Composition,Cardiopulmonary Function,and lipid metabolism in overweight or obese individuals:a meta-analysis. Chin. General Pract. 28, 335–345. 10.12114/j.issn.1007-9572.2024.0108

[B13] Dumortier M. Brandou F. Perez-Martin A. Fedou C. Mercier J. Brun J. F. (2003). Low intensity endurance exercise targeted for lipid oxidation improves body composition and insulin sensitivity in patients with the metabolic syndrome. Diabetes Metab. 29, 509–518. 10.1016/s1262-3636(07)70065-4 14631328

[B14] Ewa S. Agnieszka G. Miroslava P. Michał R. Małgorzata G. (2022). The effect of calorie restriction on the anthropometric parameters, HOMA-IR index, and lipid profile of female office workers with overweight and obesity: a preliminary study. Int. J. Occup. Med. Environ. health 35, 693–706. 10.13075/ijomeh.1896.01963 35880994 PMC10464818

[B15] Fanming K. Miaomiao Z. Jing M. Jie M. (2022). Application enlightenment of exercise and fat oxidation kinetic characteristics. Chin. J. Tissue Eng. Res. 26, 4709–4715. 10.12307/2022.843

[B16] Gallardo C. M. Hsu C. T. Gunapala K. M. Parfyonov M. Chang C. H. Mistlberger R. E. (2014). Behavioral and neural correlates of acute and scheduled hunger in C57BL/6 mice. PLoS One 9, e95990. 10.1371/journal.pone.0095990 24806659 PMC4012955

[B17] GarcíA-Prieto C. F. FernáNDEZ-Alfonso M. S. (2016). Caloric restriction as a strategy to improve vascular dysfunction in metabolic disorders. Nutrients 8, 370. 10.3390/nu8060370 27314388 PMC4924211

[B18] Guo Z. Wang M. Ying X. Yuan J. Wang C. Zhang W. (2023). Caloric restriction increases the resistance of aged heart to myocardial ischemia/reperfusion injury via modulating AMPK-SIRT(1)-PGC(1a) energy metabolism pathway. Sci. Rep. 13, 2045. 10.1038/s41598-023-27611-6 36739302 PMC9899227

[B19] Heda W. (2018). Effects of fatmax and at intensity exercise on glucose metabolism and bone metabolism in pre-diabetes middle-aged and elderly people. Genomics Appl. Biol. 37, 4231–4140. 10.13417/j.gab.037.004132

[B20] Horton J. D. Shah N. A. Warrington J. A. Anderson N. N. Park S. W. Brown M. S. (2003). Combined analysis of oligonucleotide microarray data from transgenic and knockout mice identifies direct SREBP target genes. Proc. Natl. Acad. Sci. U. S. A. 100, 12027–12032. 10.1073/pnas.1534923100 14512514 PMC218707

[B21] Husam G. Sanaa A. Scott M. Paresh D. (2018). Effect of restricted caloric intake and bariatric surgery on PCSK9 concentrations in plasma. Diabetes 67, 2093. 10.2337/db18-2093-p

[B22] Ipavec-Levasseur S. Croci I. Choquette S. Byrne N. M. Cowin G. O'Moore-Sullivan T. M. (2015). Effect of 1-h moderate-intensity aerobic exercise on intramyocellular lipids in obese men before and after a lifestyle intervention. Appl. Physiol. Nutr. Metab. 40, 1262–1268. 10.1139/apnm-2015-0258 26575100

[B23] Iwayama K. Kawabuchi R. Nabekura Y. Kurihara R. Park I. Kobayashi M. (2017). Exercise before breakfast increases 24-h fat oxidation in female subjects. PLoS One 12, e0180472. 10.1371/journal.pone.0180472 28692687 PMC5503250

[B24] JeanfréDéRIC B. Justine M. Emmanuelle V. Eric R. D. M. Jacques M. (2022). Beyond the calorie paradigm: taking into account in practice the balance of fat and carbohydrate oxidation during exercise? Nutrients 14, 1605. 10.3390/nu14081605 35458167 PMC9027421

[B25] Jiang Y. Tan S. Wang Z. Guo Z. Li Q. Wang J. (2020). Aerobic exercise training at maximal fat oxidation intensity improves body composition, glycemic control, and physical capacity in older people with type 2 diabetes. J. Exerc. Sci. and Fit. 18, 7–13. 10.1016/j.jesf.2019.08.003 31641362 PMC6796612

[B26] Jianjun L. Shuiping Z. Dong Z. Guoping L. Daoquan P. Jing L. (2023). 2023 China guidelines for lipid management. J. geriatric Cardiol. JGC 20, 621–663. 10.26599/1671-5411.2023.09.008 PMC10568545 37840633

[B27] Kiesswetter E. (2017). Nutrition and sarcopenia. Osteologie 26, 28–31. 10.1055/s-0037-1622078

[B28] Lagace T. A. (2014). PCSK9 and LDLR degradation: regulatory mechanisms in circulation and in cells. Curr. Opin. Lipidol. 25, 387–393. 10.1097/MOL.0000000000000114 25110901 PMC4166010

[B29] Luna-Castillo K. P. Olivares-Ochoa X. C. HernáNDEZ-Ruiz R. G. Llamas-Covarrubias I. M. RodríGUEZ-Reyes S. C. Betancourt-NúñEZ A. (2022). The effect of dietary interventions on hypertriglyceridemia: from public health to molecular nutrition evidence. Nutrients 14, 1104. 10.3390/nu14051104 35268076 PMC8912493

[B30] Ma J. Zheng Y. Sun F. Fan Y. Fan Y. Su X. (2023). Research progress in the correlation between SREBP/PCSK9 pathway and lipid metabolism disorders induced by antipsychotics. Univ. Med. Sci. 48, 1529–1538. 10.11817/j.issn.1672-7347.2023.230029 PMC10929898 38432882

[B31] Martin C. K. Bhapkar M. Pittas A. G. Pieper C. F. Das S. K. Williamson D. A. (2016). Effect of calorie restriction on mood, quality of life, sleep, and sexual function in healthy nonobese adults: the CALERIE 2 randomized clinical trial. JAMA Intern Med. 176, 743–752. 10.1001/jamainternmed.2016.1189 27136347 PMC4905696

[B32] Maunder E. Plews D. J. Kilding A. E. (2018). Contextualising maximal fat oxidation during exercise: determinants and normative values. Front. Physiol. 9, 599. 10.3389/fphys.2018.00599 29875697 PMC5974542

[B33] Mitchell S. J. Madrigal-Matute J. Scheibye-Knudsen M. Fang E. Aon M. GonzáLEZ-Reyes J. A. (2016). Effects of sex, strain, and energy intake on hallmarks of aging in mice. Cell Metab. 23, 1093–1112. 10.1016/j.cmet.2016.05.027 27304509 PMC4911707

[B34] NapoleãO A. Fernandes L. Miranda C. Marum A. P. (2021). Effects of calorie restriction on health span and insulin resistance: classic calorie restriction diet vs. Ketosis-inducing diet. Nutrients 13, 1302. 10.3390/nu13041302 33920973 PMC8071299

[B35] Ochner C. N. Barrios D. M. Lee C. D. Pi-Sunyer F. X. (2013). Biological mechanisms that promote weight regain following weight loss in obese humans. Physiol. Behav. 120, 106–113. 10.1016/j.physbeh.2013.07.009 23911805 PMC3797148

[B36] Peng Z. H. Yang M. E. I. Zhou HUIMIN Liu XIAOLI Wang CHENGKE (2022). The effects of 12-week FATmax intensity exercise on blood glucose, blood lipids and liver function in obese non-alcoholic fatty liver patients. Genomics Appl. Biol. 41, 648–658. 10.13417/j.gab.041.000648

[B37] Pikkemaat M. Woodward M. Geijerstam P. A. Harrap S. Hamet P. Mancia G. (2024). Lipids and apolipoproteins and the risk of vascular disease and mortality outcomes in women and men with type 2 diabetes in the ADVANCE study. Diabetes, Obes. and metabolism 26, 5669–5680. 10.1111/dom.15935 39256935

[B38] Purdom T. Kravitz L. Dokladny K. Mermier C. (2018). Understanding the factors that effect maximal fat oxidation. J. Int. Soc. Sports Nutr. 15, 3. 10.1186/s12970-018-0207-1 29344008 PMC5766985

[B39] RäMö J. Jurgens S. J. Kany S. Choi S. H. Wang X. Smirnov A. N. (2024). Rare genetic variants in LDLR, APOB, and PCSK9 are associated with aortic stenosis. Circulation 150, 1767–1780. 10.1161/CIRCULATIONAHA.124.070982 39222019 PMC11915876

[B40] Ramos-Campo D. J. Rojo-Tirado M. Benito-Peinado P. J. (2024). Investigating the impact of exercise type combined with caloric restriction on quality of life-The PRONAF study. Physiol. Behav. 283, 114614. 10.1016/j.physbeh.2024.114614 38866299

[B41] Rubio-Valles M. Amaro-Gahete F. J. Creasy S. A. Ramos-JiméNEZ A. PéREZ-LeóN J. A. CháVEZ-Guevara I. A. (2024). Circadian regulation of fatty acid metabolism in humans: is there evidence of an optimal time window for maximizing fat oxidation during exercise? Sports Med. 10.1007/s40279-024-02154-6 39681771

[B42] Sanchez-Delgado G. Alcantara J. M. A. Ortiz-Alvarez L. Xu H. Martinez-Tellez B. Labayen I. (2018). Reliability of resting metabolic rate measurements in young adults: impact of methods for data analysis. Clin. Nutr. 37, 1618–1624. 10.1016/j.clnu.2017.07.026 28826698

[B43] Senesi P. Ferrulli A. Luzi L. Terruzzi I. (2021). Diabetes mellitus and cardiovascular diseases: nutraceutical interventions related to caloric restriction. Int. J. Mol. Sci. 22, 7772. 10.3390/ijms22157772 34360538 PMC8345941

[B44] Sijie T. Ping D. Wanting Z. Jiaqi P. Jianxiong W. (2018). Exercise training at maximal fat oxidation intensity for older women with type 2 diabetes. Int. J. sports Med. 39, 374–381. 10.1055/a-0573-1509 29564847

[B45] Smith D. L. Yang Y. Mestre L. M. Henschel B. Parker E. Dickinson S. (2024). Impact of sustained calorie restriction and weight cycling on body composition in high-fat diet-fed male and female C57BL/6J mice. Obes. (Silver Spring, Md.) 32, 959–968. 10.1002/oby.24015 PMC11145641 38600047

[B46] Son D. H. Kwon Y. J. Lee H. S. Kim H. M. Lee J. W. (2021). Effects of a calorie-restricted mediterranean-style diet on plasma lipids in hypercholesterolemic south Korean patients. Nutrients 13, 3393. 10.3390/nu13103393 34684393 PMC8539389

[B47] Soutar A. K. (2011). Unexpected roles for PCSK9 in lipid metabolism. Curr. Opin. Lipidol. 22, 192–196. 10.1097/MOL.0b013e32834622b5 21494143

[B48] Sun H. Samarghandi A. Zhang N. Yao Z. Xiong M. Teng B. B. (2012). Proprotein convertase subtilisin/kexin type 9 interacts with apolipoprotein B and prevents its intracellular degradation, irrespective of the low-density lipoprotein receptor. Arterioscler. Thromb. Vasc. Biol. 32, 1585–1595. 10.1161/ATVBAHA.112.250043 22580899

[B49] Tanja K. Mia G. Geoffrey L. Willy W. A L. T. (2013). Low density lipoprotein binds to proprotein convertase subtilisin/kexin type-9 (PCSK9) in human plasma and inhibits PCSK9-mediated low density lipoprotein receptor degradation. J. Biol. Chem. 288, 8279–8288. 10.1074/jbc.m112.421370 23400816 PMC3605646

[B50] Tan S. Wang J. Cao L. (2016). Exercise training at the intensity of maximal fat oxidation in obese boys. Appl. Physiol. Nutr. Metab. 41, 49–54. 10.1139/apnm-2015-0174 26701116

[B51] Thonusin C. Pantiya P. Sumneang N. Chunchai T. Nawara W. Arunsak B. (2022). Effectiveness of high cardiorespiratory fitness in cardiometabolic protection in prediabetic rats. Mol. Med. 28, 31. 10.1186/s10020-022-00458-9 35272616 PMC8908596

[B52] Tianxiao L. Dong Z. Yue Q. (2022). Global trends in the epidemiology and management of dyslipidemia. J. Clin. Med. 11, 6377. 10.3390/jcm11216377 36362605 PMC9656679

[B53] Vincenzo Q. Irma B. Massimiliano B. Martina I. Laura C. M. Carlo M. (2023). PCSK9 inhibitors in cancer patients treated with immune-checkpoint inhibitors to reduce cardiovascular events: new frontiers in cardioncology. Cancers 15, 1397. 10.3390/cancers15051397 36900189 PMC10000232

[B54] Wang Y. Y. Tian F. Qian X. L. Ying H. M. Zhou Z. F. (2024). Effect of 5:2 intermittent fasting diet versus daily calorie restriction eating on metabolic-associated fatty liver disease—a randomized controlled trial. Front. Nutr. 11, 1439473. 10.3389/fnut.2024.1439473 39229586 PMC11368853

[B55] Wang B. Tontonoz P. (2018). Liver X receptors in lipid signalling and membrane homeostasis. Nat. Rev. Endocrinol. 14, 452–463. 10.1038/s41574-018-0037-x 29904174 PMC6433546

[B56] Xie Y. Gu Y. Li Z. He B. Zhang L. (2024). Effects of different exercises combined with different dietary interventions on body composition: a systematic review and network meta-analysis. Nutrients 16, 3007. 10.3390/nu16173007 39275322 PMC11397086

[B57] Yingying L. Li P. Chenchao B. (2018). Effects of different test programs on the maximal fat oxidation in young females. Chin. J. Tissue Eng. Res. 22, 5145–5149. 10.3969/j.issn.2095-4344.0393

[B58] Yong Z. Zhijun L. (2013). Research on fat oxidation kinetics and exercise intensity eliciting maximal fat oxidation in endurance trained and untrained women. China Sport Sci. 33, 61–68. 10.3969/j.issn.2095-4344.0393

[B59] Yu W. Qin J. Chen C. Fu Y. Wang W. (2018). Moderate calorie restriction attenuates age-associated alterations and improves cardiac function by increasing SIRT1 and SIRT3 expression. Mol. Med. Rep. 18, 4087–4094. 10.3892/mmr.2018.9390 30132522

[B60] Zhu W. Chen L. Zhang J. (2020). Advances in lipocalin and lipocalin receptor based intervention pathways for metabolic syndrome. Clin. Educ. General Pract. 18, 1110–1113. 10.13558/j.cnki.issn1672-3686.2020.012.015

